# Spinal Anesthesia for Open Appendectomy in a Patient With Cystic Fibrosis and Coagulation Abnormalities: A Case of Tailored Anesthetic Management

**DOI:** 10.7759/cureus.72824

**Published:** 2024-11-01

**Authors:** Leonor Lemos, Henrique Guimarães, Eduardo Tavares, Tiago Miguel Cardoso, António Costa, Cristina Poiarez

**Affiliations:** 1 Anesthesiology and Critical Care, Centro Hospitalar Universitário de Santo António, Porto, PRT

**Keywords:** anesthetic management, cystic fibrosis, open appendectomy, respiratory complications, spinal anesthesia

## Abstract

Cystic fibrosis (CF) is a genetic disorder that primarily affects the respiratory and gastrointestinal systems, often leading to significant perioperative challenges due to compromised lung function, recurrent infections, and chronic respiratory failure. Managing anesthesia in patients with CF requires careful consideration, particularly because of the increased risk of respiratory complications with general anesthesia (GA). Neuraxial anesthesia, such as spinal anesthesia, presents an alternative that can reduce the likelihood of postoperative pulmonary issues, including respiratory depression, hypoxemia, and atelectasis. However, spinal anesthesia is not without risk, particularly in the presence of coagulation abnormalities, which must be considered.

We present the case of a 26-year-old male with severe CF, complicated by chronic respiratory failure and recurrent infections, who presented with acute appendicitis. The patient, with a history of bronchiectasis and chronic colonization by multidrug-resistant organisms, had stable but significantly impaired respiratory function, with a forced expiratory volume in one second (FEV1) of 1.62 L (38% predicted). He had a history of multiple hospital admissions for exacerbations of lung disease, none of which required mechanical ventilation or intensive care, with the most recent admission occurring 13 months prior to this event.

Preoperative coagulation studies revealed an elevated international normalized ratio (INR) of 1.52 (normal range 0.8-1.2) and an activated partial thromboplastin time (APTT) of 38.1 seconds (normal <29.4 seconds), for which the patient received vitamin K without improvement. Despite these abnormalities, a thorough preoperative assessment and multidisciplinary discussion led to the decision to proceed with spinal anesthesia, carefully weighing the risks and benefits. An initial spinal anesthetic attempt with 2.0 mL of hyperbaric bupivacaine 0.5% (10 mg) and 2.5 mcg of sufentanil was administered at the L3-L4 interspace using a 27G Whitacre needle. Despite confirmed cerebrospinal fluid flow before injection, no sensory or motor block occurred, requiring an additional spinal injection. A second dose of 2.0 mL of hyperbaric bupivacaine 0.5% was administered at the L2-L3 interspace without sufentanil. A satisfactory sensory block to the T6 level was eventually obtained, allowing the appendectomy to proceed without intraoperative complications. Postoperatively, the patient was closely monitored in a high-dependency unit. He maintained stable respiratory function and experienced a smooth recovery, with minimal opioid use to avoid respiratory depression. The patient was discharged on the fifth postoperative day without respiratory or other anesthesia-related complications.

This case highlights the importance of individualized care in CF patients undergoing surgery. Neuraxial anesthesia, when carefully planned and executed, can offer a safer alternative to GA by minimizing respiratory risks. However, the presence of coagulation abnormalities requires a detailed risk-benefit analysis, multidisciplinary collaboration, and vigilant intraoperative and postoperative care to ensure patient safety and optimize outcomes.

## Introduction

Cystic fibrosis (CF) is an autosomal-recessive disorder caused by mutations in a gene located on the long arm of chromosome 7 that encodes the CF transmembrane conductance regulator (CFTR) protein, which is expressed in many epithelial cells [1]. Abnormal airway surface liquid results in recurrent lower respiratory tract infections and airway remodeling, leading to increased airway resistance, gas trapping, ventilation-perfusion mismatching, and increased work of breathing [2]. In severe cases, patients may experience hypoxemia, hypercapnia, and chronic respiratory failure [3].

With an estimated incidence between 1 in 3,000 and 1 in 6,000, the epidemiological profile of CF has changed significantly in recent decades, evolving from a primarily pediatric condition to one that increasingly affects adults [4]. As life expectancy improves, an increasing number of CF patients are expected to require surgery.

Anesthetic management in patients with CF requires meticulous planning. The anesthetic plan should focus on maximizing the clearance of viscous respiratory secretions and minimizing the risk of postoperative respiratory complications [5]. Where possible, neuraxial or regional anesthesia techniques should be considered to avoid airway instrumentation and mechanical ventilation [3].

Neuraxial techniques may offer substantial benefits, including a reduced risk of respiratory depression, pneumonia, and atelectasis during the perioperative period [6]. Nonetheless, these techniques are not without their own risks, particularly in patients with coagulopathy.

This case report describes the complex management of anesthesia in a patient with acute appendicitis who had a history of severe CF and coagulation abnormalities.

## Case presentation

A 26-year-old male, weighing 48 kg and measuring 169 cm in height, with a history of CF caused by the G542X/Q110P mutation, presented to the Emergency Department with diffuse abdominal pain predominantly in the upper quadrants, anorexia, a single episode of nonbilious vomiting, and decreased bowel movements over two days. The patient denied fever and other systemic symptoms. From the abdominal examination, it is worth highlighting the presence of bowel sounds in all four quadrants and diffuse pain on palpation that appeared to worsen with decompression at McBurney’s point. There were no signs of peritonitis.

Medical history

The patient’s CF was diagnosed at four months of age and has been complicated by severe respiratory involvement, including bronchiectasis, recurrent hemoptysis, and chronic colonization by multiple drug-resistant organisms, such as *Pseudomonas aeruginosa*, methicillin-sensitive *Staphylococcus aureus* (MSSA), *Stenotrophomonas maltophilia*, and *Achromobacter xylosoxidans* ssp. *denitrificans*. He has undergone multiple hospital admissions, averaging one to two per year, due to pulmonary exacerbations, although none required mechanical ventilation or intensive care. Notably, he was hospitalized five times in the past five years, with the most recent admission occurring 13 months prior to this episode. He was awaiting an evaluation for lung transplantation. His long-term treatment is outlined in Table 1.

**Table 1:** Patient's usual medication

Drug	Dosage
Azithromycin	500 mg three days a week
Aztreonam (inhaled)	75 mg three times a day
Dornase alfa	2.5 mg daily
Liposoluble vitamins A, D, E, K	Two capsules per day
Oxygen	1 L/min overnight
Pancreatin	15,000 IU daily
Pantoprazole	20 mg daily
Sulfamethoxazole/trimethoprim	800/160 mg three days a week
Tobramycin (inhaled)	112 mg twice daily
Ursodeoxycholic acid	250 mg twice daily

**Figure 1:**
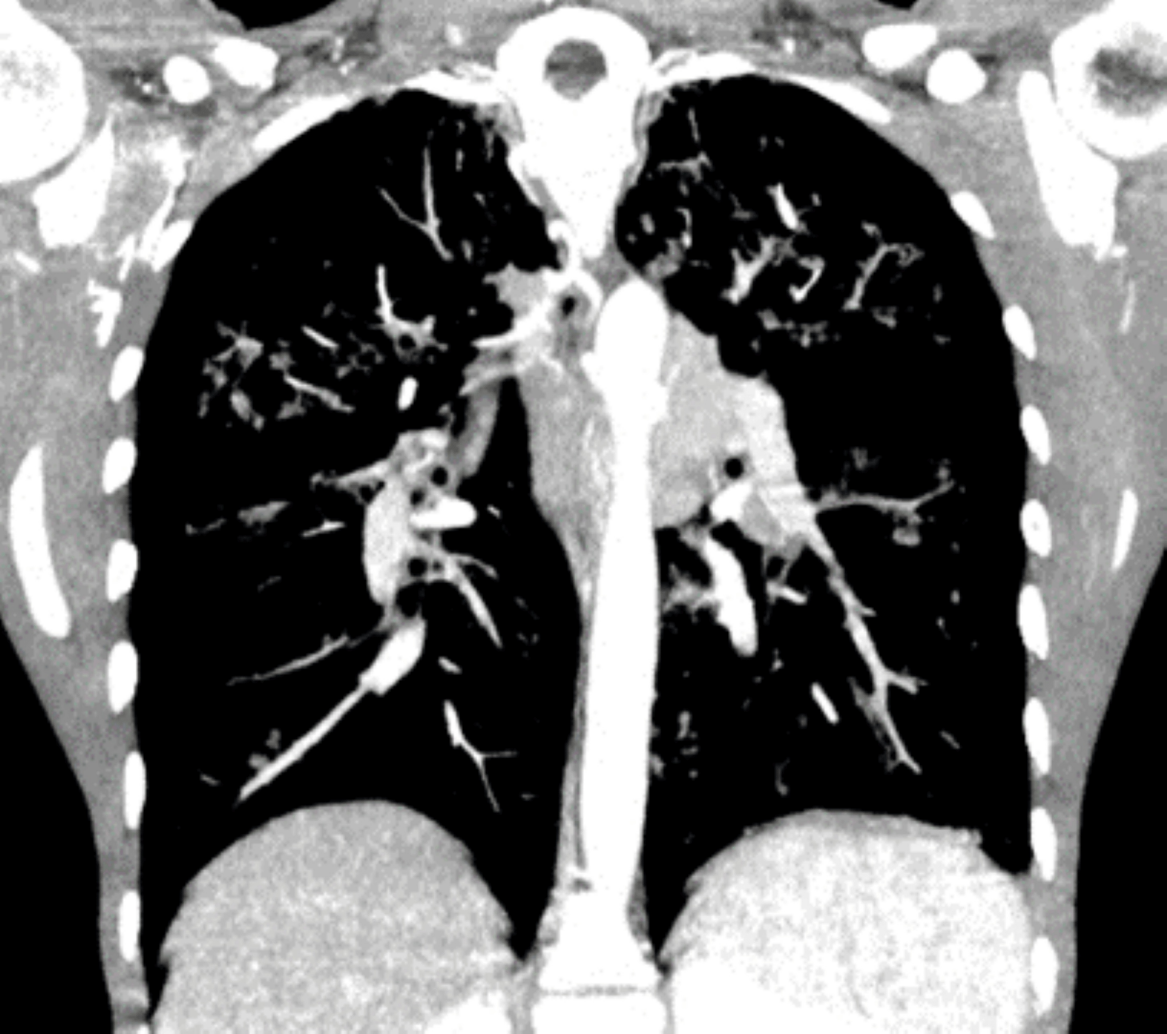
Patient's last CT scan (March 2023) CT scan showed multiple bronchiectasis features dispersed across all pulmonary lobes, ectasia of the bronchial arteries, and multiple hilar and mediastinal adenomegalies, among other findings. CT: Computed tomography

Of note, inhaled tobramycin and aztreonam are taken in alternating courses of 28 days. Additionally, hypertonic saline, a short-acting β-2 agonist, and N-acetylcysteine were used only as needed. He was on low-flow nocturnal oxygen therapy at 1 L/min and had a baseline oxygen saturation of 93% on room air. Recent thoracic imaging (Figure 1) revealed extensive bronchiectasis, atelectasis, and a persistent micronodular infiltrate.

Pulmonary function tests demonstrated severe obstructive and restrictive lung disease, with a forced expiratory volume in one second (FEV1) of 1.62 L (38% predicted). His last abdominal ultrasound revealed hepatomegaly (17.8 cm in the midclavicular line) and pancreatic calcifications. The latest routine examinations revealed normal levels of albumin, transaminases, and coagulation parameters.

Initial assessment

The patient’s respiratory status had been stable, with a slight increase in sputum production over the past month, managed effectively with chest physiotherapy. His exercise tolerance was moderately reduced, with dyspnea on exertion. A blood gas analysis upon admission revealed a PaO₂ of 62 mmHg, consistent with baseline values. On pulmonary auscultation, diffuse crackles were present throughout both lung fields.

Laboratory findings included leucocytosis with a white blood cell count of 19,300/mm³, predominantly neutrophilic; an elevated C-reactive protein level of 83 mg/L; and hemoglobin of 13.6 g/dL. Abdominal computed tomography showed features of chronic pancreatitis with pancreatic lipomatosis, generalized small bowel dilatation with fecal impaction in the terminal ileum, moderate peritoneal effusion, and biliary tract ectasia.

Preoperative coagulation studies showed an activated partial thromboplastin time (APTT) of 38.1 seconds and an international normalized ratio (INR) of 1.52, for which the patient received 10 mg of intravenous vitamin K. Tests were repeated six hours later with no improvement. The platelet count was 735,000/mm³. Complete laboratory results are described in Table 2.

**Table 2:** Pre-operative study - blood test results

Test	Result	Normal range
White blood cells	19,300/mm^3^	4,000-11,000/mm^3^
Neutrophils	85%	40-75%
Hemoglobin	13.6 g/dL	13.5-17.5 g/dL (male)
Platelets	735,000/mm^3^	150,000-450,000/mm^3^
Amylase	<3 U/L	0-53 U/L
Lipase	6 U/L	30-190 U/L
Glucose	94 mg/dL	70-100 mg/dL
Creatinine	0.56 mg/dL	0.6-1.2 mg/dL
Urea	20 mg/dL	10-50 mg/dL
Total bilirubin	0.86 mg/dL	0.2-1 mg/dL
Aspartate aminotransferase (AST) (TGO)	9 U/L	10-40 U/L
Alanine aminotransferase (ALT) (TGP)	7 U/L	7-56 U/L
Alkaline phosphatase	89 U/L	40-129 U/L
Gamma-glutamyl transferase (GGT)	15 U/L	10-66 U/L
Lactate dehydrogenase (LDH)	284 U/L	135-225 U/L
Albumin	3.72 g/dL	3.5-5 g/dL
C-reactive protein	83 mg/L	0-5 mg/L
Total proteins	8.6 g/dL	6-7.3 g/dL
Sodium	135 mmol/L	135-145 mmol/L
Potassium	4.6 mmol/L	3.5-5.1 mmol/L
Chloride	96 mmol/L	96-106 mmol/L
Activated partial thromboplastin time	38.1 s	<29.4 s
Prothrombin time	16.8 s	<11.4 s
International normalized ratio (INR)	1.52	0.8-1.2

Anesthetic management

American Society of Anesthesiologists (ASA) standard monitoring, including non-invasive blood pressure, pulse oximetry, and electrocardiography, was used. With the patient in right lateral decubitus, a mixture of 2.0 mL of hyperbaric bupivacaine 0.5% (10 mg) and 2.5 mcg of sufentanil (total volume 2.5 mL) was administered at the L3-L4 interspace using a 27G Whitacre needle. A free flow of clear cerebrospinal fluid was observed in all four quadrants prior to injection, and the patient was then placed in the supine position. No sensory or motor block occurred at any level, and there were no changes in hemodynamic parameters in the subsequent 30 minutes. Given the failed spinal, the decision was made to perform a second spinal injection. With the patient in the same position and using a similar needle, a further 2.0 mL of hyperbaric bupivacaine 0.5% from a new vial was administered at a higher interspace (L2-L3), this time without sufentanil. After the second injection, a satisfactory sensory block up to T6 was achieved within 10 minutes. The procedure was completed without any intraoperative complications. The patient’s hemodynamics remained stable, with no evidence of respiratory distress or desaturation during the operation.

Outcome and follow-up

Postoperatively, the patient was closely monitored in a high-dependency unit. He did not require mechanical ventilation and maintained adequate oxygenation on low-flow oxygen therapy. Pain was well controlled with fixed-schedule paracetamol and nonsteroidal anti-inflammatory drugs (NSAIDs), with no need for opioid rescue analgesia, reducing the risk of respiratory depression. The patient had a smooth recovery and was discharged from the hospital on the fifth postoperative day, with no exacerbation of his respiratory condition.

## Discussion

In patients with CF, lung function deteriorates despite various pharmacologic treatments, and hypercapnic respiratory failure often develops as the disease progresses [7]. Consequently, these patients are at heightened risk of perioperative complications, particularly when general anesthesia (GA) is used. Given the patient’s compromised respiratory function, any procedure requiring mechanical ventilation posed a significant risk of exacerbation and prolonged respiratory failure. Considering this, performing the surgery under neuraxial anesthesia appeared to be the optimal option, as it would minimize respiratory depression, avoid intubation, and reduce the risk of postoperative pulmonary complications. After discussion with the surgical team, an open appendectomy was chosen, as this option was deemed ideal for performing neuraxial anesthesia.

Routine coagulation screening is not recommended prior to a procedure, as it does not indicate the bleeding risk, nor does a normal screen exclude a bleeding disorder [8,9]. However, given the patient’s acute appendicitis, prolonged antibiotic use, malnutrition (BMI < 18 kg/m²), and chronic pancreatitis - all of which may contribute to coagulopathy - requesting and awaiting the results of these studies before proceeding with a high-risk hemorrhagic procedure such as spinal anesthesia was recommended [8]. Additionally, per the British Society for Haematology and European Society of Anaesthesiology guidelines, a detailed bleeding history was taken. The patient denied excessive or prolonged bleeding, and there were no known coagulation disorders in the family. According to electronic medical records from follow-up visits, there was no history of coagulation-related issues or liver failure. The patient also denied the use of anticoagulants or antiplatelet medications.

The altered coagulation study, with elevated INR and APTT, presented a challenge in the decision-making process regarding the performance of spinal anesthesia. This also precluded the choice of combined spinal-epidural anesthesia, a technique that could have extended the duration of the block and been used for postoperative pain control.

Given the coagulation study results, the Immunohematology Department was consulted. Due to the elevated INR in a patient with risk factors for vitamin K deficiency, they recommended the administration of phytomenadione. No other interventions, such as the use of fresh frozen plasma, were suggested, as these were unlikely to correct the prothrombin time (PT)/INR in this context [10]. Moreover, according to the literature, the utility of PT and APTT as screening tools for coagulopathy is limited, as they often reflect in vitro results that do not account for the complex process of hemostatic rebalancing that occurs in acutely ill patients [11]. The relevance of performing rotational thromboelastometry (ROTEM) in our operating room was also questioned. However, given its limitations and lack of evidence in predicting hemorrhage or the need for transfusion components in patients without documented liver disease, it was considered that it would not provide additional pertinent information to the presented clinical scenario [12].

According to the Nordic guidelines for neuraxial blocks in disturbed hemostasis from the Scandinavian Society of Anaesthesiology and Intensive Care Medicine, if a reduced risk of serious postoperative complications is expected by administering a central neuraxial block, some increased risk of spinal hematoma can be accepted. These guidelines suggest that, where there is potential benefit from neuraxial blocks in terms of morbidity reduction, INR levels of less than 1.8 for single-shot spinal anesthesia may be acceptable [13].

Given the risk associated with the procedure, we considered that having an experienced operator perform the technique was appropriate in this context. The need to repeat the procedure following the failure of the initial attempt was also thoroughly discussed. Options included repeating the spinal anesthesia, providing pain relief through opioids or sedation, or switching to GA. Given the circumstances previously discussed, we decided that spinal anesthesia was the most appropriate rescue technique.

The decision to admit the patient to an Intermediate Care Unit was based on the underlying condition and the need for close monitoring for possible complications, particularly epidural/spinal hematoma, which, although rare, was one of the most concerning potential complications in this case.

## Conclusions

This case underscores the importance of individualized care and close collaboration with the surgical team to determine the safest and most effective treatment strategy for patients with complex conditions, such as those with severe respiratory diseases. When the optimal anesthetic technique is not immediately clear, a multidisciplinary discussion is essential to select the approach that minimizes risk. In this instance, the risk of complications was increased regardless of the anesthetic strategy. The successful use of spinal anesthesia, despite its inherent challenges, highlights its potential as a viable alternative to GA for low abdominal surgery in patients with compromised pulmonary function, such as those with CF. The case also emphasizes that meticulous preoperative assessment, vigilant intraoperative monitoring, and thorough postoperative care are critical for optimizing outcomes and managing the increased risk of complications inherent in such complex scenarios.
